# Hybrid Cellulose–Basalt Polypropylene Composites with Enhanced Compatibility: The Role of Coupling Agent

**DOI:** 10.3390/molecules25194384

**Published:** 2020-09-24

**Authors:** Claudia Sergi, Francesca Sbardella, Matteo Lilli, Jacopo Tirillò, Andrea Calzolari, Fabrizio Sarasini

**Affiliations:** 1 Department of Chemical Engineering Materials Environment & UdR INSTM, Sapienza-Università di Roma, Via Eudossiana 18, 00184 Roma, Italy; claudia.sergi@uniroma1.it (C.S.); francesca.sbardella@uniroma1.it (F.S.); matteo.lilli@uniroma1.it (M.L.); 2 IMAST S.c.ar.l.-Technological District on Engineering of Polymeric and Composite Materials and Structures, Piazza Bovio 22, 80133 Napoli, Italy; 3 ITW Test and Measurement Italy, Via Airauda 12, 10044 Pianezza (TO), Italy; Andrea_Calzolari@instron.com

**Keywords:** polymer-matrix composites, hybrid, basalt fibers, microcrystalline cellulose, polypropylene, interface, mechanical properties

## Abstract

This study deals with the development and optimization of hybrid composites integrating microcrystalline cellulose and short basalt fibers in a polypropylene (PP) matrix to maximize the mechanical properties of resulting composites. To this aim, the effects of two different coupling agents, endowed with maleic anhydride (MA-g(grafted)-PP) and acrylic acid (AA-g-PP) functionalities, on the composite properties were investigated as a function of their amount. Tensile, flexural, impact and heat deflection temperature tests highlighted the lower reactivity and effectiveness of AA-g-PP, regardless of reinforcement type. Hybrid formulations with basalt/cellulose (15/15) and with 5 wt. % of MA-g-PP displayed remarkable increases in tensile strength and modulus, flexural strength and modulus, and notched Charpy impact strength, of 45% and 284%, 97% and 263%, and 13%, in comparison with neat PP, respectively. At the same time, the thermo-mechanical stability was enhanced by 65% compared to neat PP. The results of this study, if compared with the ones available in the literature, reveal the ability of such a combination of reinforcements to provide materials suitable for automotive applications with environmental benefits.

## 1. Introduction

Natural fiber-reinforced composites owe their use to the increasing demand for lightweight materials endowed with a reduced environmental footprint. This is particularly true in the automotive sector, where their inherent properties, such as biodegradability, being lightweight and low-cost, and life-cycle superiority compared to synthetic fiber-reinforced composites, are well appreciated. Currently, plastics account for 10–12% of the total weight of a vehicle, and it is roughly calculated that 75% of fuel consumption is dictated by the vehicle’s weight [1,2]. A decrease of 10% in a vehicle’s weight is estimated to result in a 6–8% reduction in fuel consumption [1]. These considerations, coupled with a stricter legislation, have triggered the wide acceptance of natural fiber composites in the automotive sector, mainly as nonstructural interior parts, such as indoor panels, dashboards, seat backs, headliners and storage bins.

The use of cellulose fibers for addressing the fuel economy standards of vehicle’s manufacturers is unfortunately hampered by some drawbacks, such as lower modulus and strength, inferior moisture resistance and lower thermal stability compared to glass fiber-reinforced composites, combined with an inherent incompatibility with hydrophobic matrices [3,4]. Fiber hybridization is a widely recognized strategy to improve the performance of natural fiber composites, especially with inorganic fibers. In this regard, glass fibers have proven to be quite successful in providing composites with a more balanced set of properties [5,6,7]. An alternative to glass fibers is represented by basalt fibers, which are natural fibers of mineral origin exhibiting mechanical properties comparable, if not superior, to those of E-glass fibers with improved thermal and chemical resistance [8]. Composites based on basalt fibers have been widely investigated in the literature [9,10,11,12,13,14,15], and their positive effect in combination with natural fibers has been confirmed [16,17,18,19].

Currently, the most common thermoplastic matrices used in the automotive sector are polypropylene, polyamide 6, polyamide 66, polyoxymethylene (polyacetal) and polyethylene [20]. In particular, polypropylene (PP) is extensively used because it is widely available, inexpensive, and easily processable with a good combination of chemical and moisture resistance. In this regard, hybrid composites made with cellulose and basalt fibers feature benefits in weight reduction and sustainability, with mechanical properties that can be tailored to become suitable for more demanding applications in the automotive sector, such as those under-the-hood, where high tensile and flexural properties and heat resistance are needed.

In this context, the aim of this work was to develop hybrid composites addressing the effect of combining microcrystalline cellulose with basalt fibers in a polypropylene matrix, with a view to enhancing the biobased amount by decreasing the inorganic reinforcement, despite being renewable in nature. An envisaged major technical barrier is the well-known incompatibility of a hydrophobic polymer, like PP, with polar cellulose and basalt fibers. The resulting poor interfacial adhesion has been the subject of extensive investigations, and one relatively simple and effective strategy to face this issue involves matrix modification with a polymer compatibilizer.

To foster fiber/matrix adhesion, a standard coupling agent is maleic anhydride-grafted polypropylene (MA-g-PP), which involves a reaction of maleic anhydride with the hydroxyl groups available on cellulosic and basalt fibers, while the propylene part reacts with the base polypropylene matrix during melt compounding [20]. Botev et al. [21] tested several amounts of basalt fibers in a PP matrix, and reported a decrease in tensile yield strength and an increase in Izod impact strength with increasing amounts of basalt fibers. The same properties were found to increase with increasing amounts of MA-g-PP, which was introduced in different concentrations, ranging from 0.5 to 2 wt. %. Spoljaric et al. [22] investigated the dynamic mechanical behavior, thermal stability and crystallization of PP-based composites reinforced with microcrystalline cellulose. Cellulose was subjected to different surface treatments, including silicone oil, stearic acid and alkyltitanate with or without the addition of MA-g-PP. The coupling agent improved the thermal stability of PP, increased the storage modulus and decreased the creep deformation of the composites due to an enhanced compatibility. Despite the well documented positive effects on natural fibers and inorganic reinforcements, the effectiveness of the coupling agent depends on several factors, including the graft level, viscosity, fiber content and type [23]. This means that the properties of PP-based composites can be fine-tailored to meet diverse requirements, but this is still an up-to-date and unsolved issue, even for glass-based polypropylene composites [24], because MA-g-PP can increase the performance of PP-based composites only if it is able to wet the reinforcement, chemically react with it and interdiffuse within the polymer bulk [25]. The adhesion at the fiber interface is dictated by the surface energies of the materials and the chemical functional groups of the matrix and the fiber sizing. In this regard, several functionalities have been investigated [26] in addition to the design of specific sizings. Recently, Ralph et al. discussed the effects of four innovative sizings for basalt fibers optimized for PP, both at the fiber/matrix scale [27] and at the composite scale [28]. One sizing, by decreasing the polar component of the fiber’s surface energy, was found to be particularly effective in increasing the tensile and flexural properties of the resulting composites at the expense of the impact strength. No detailed information about the sizings were provided because of their proprietary nature.

In the present work, innovative hybrid composites based on basalt fibers and microcrystalline cellulose were developed in a PP matrix to optimize the overall composite properties. To this end, two different coupling agents were used, namely a maleic anhydride modified-polypropylene (MA-g-PP) and an acrylic acid-modified polypropylene (AA-g-PP). The mechanical (tensile, flexural and impact), thermal (heat deflection temperature) and morphological properties were evaluated in terms of type and amount (2, 5 and 8 wt. %) of coupling agent, as well as relative proportion of fibers, highlighting their feasibility for automotive applications. 

## 2. Results and Discussion

### 2.1. Effect of Coupling Agent Type and Amount on Neat PP

**Figure 1:**
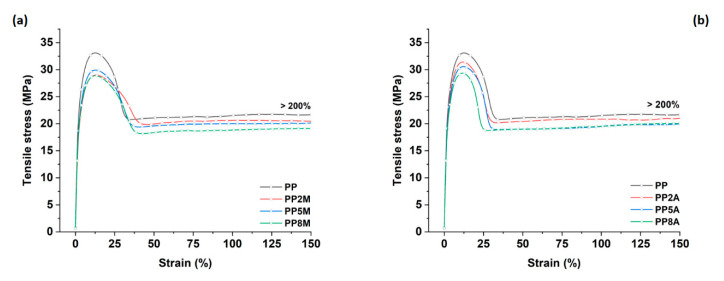
Tensile stress vs. strain curves for neat PP as a function of coupling agent type and amount: (**a**) MA-g-PP and (**b**) AA-g-PP.

**Figure 2:**
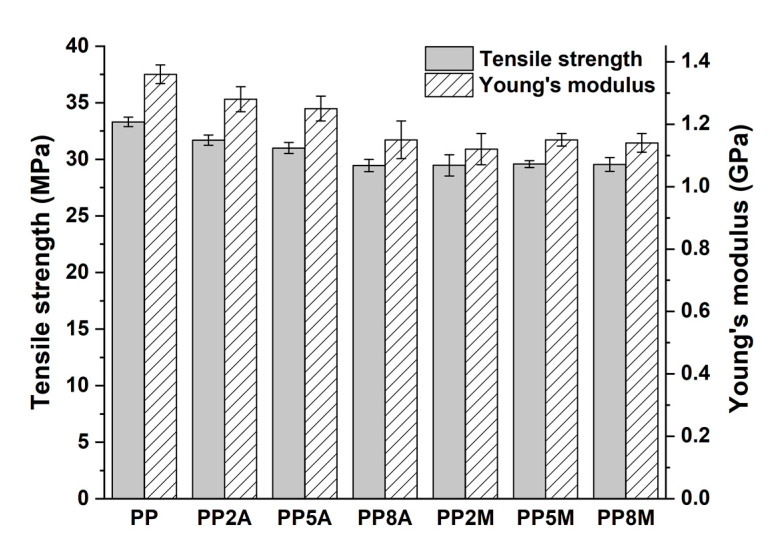
Tensile strength and Young’s modulus as a function of coupling agent type and amount.

To discriminate the effect of coupling agent type and content on the original matrix, a detailed mechanical and thermal characterization was carried out. Figure 1 shows the typical tensile stress vs. strain curves of neat PP modified with different amounts of both coupling agents, whereas the mechanical properties calculated from the curves are summarized in Figure 2.

**Figure 3:**
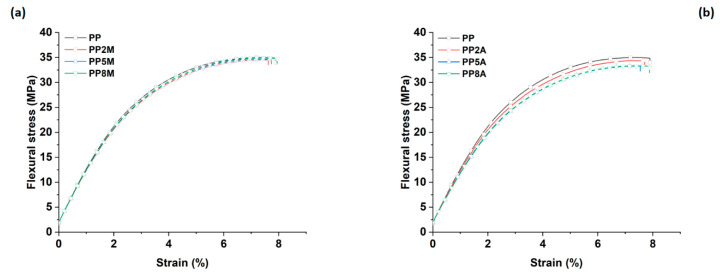
Flexural stress vs. strain curves for neat PP as a function of coupling agent type and amount: (**a**) MA-g-PP and (**b**) AA-g-PP.

**Figure 4:**
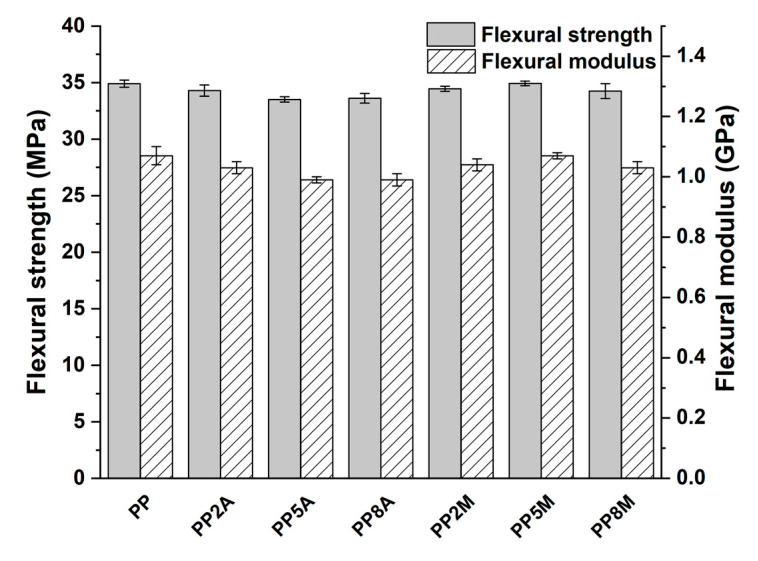
Flexural strength and modulus as a function of coupling agent type and amount.

**Figure 5:**
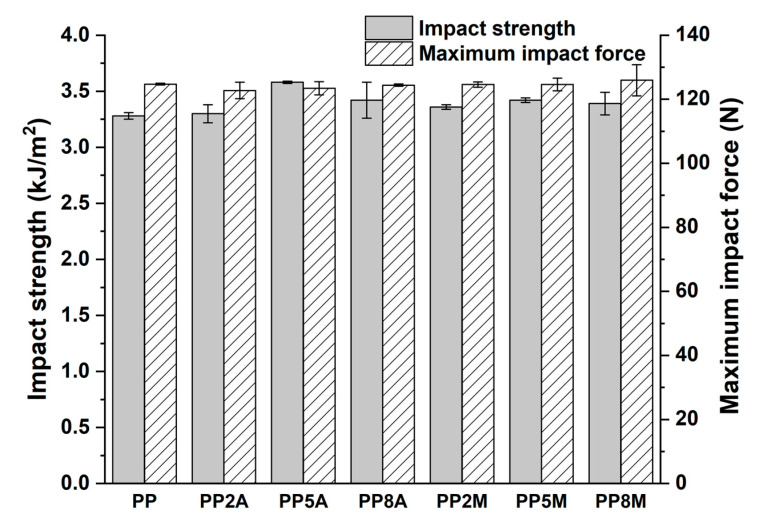
Charpy impact strength as a function of coupling agent type and amount.

**Figure 6:**
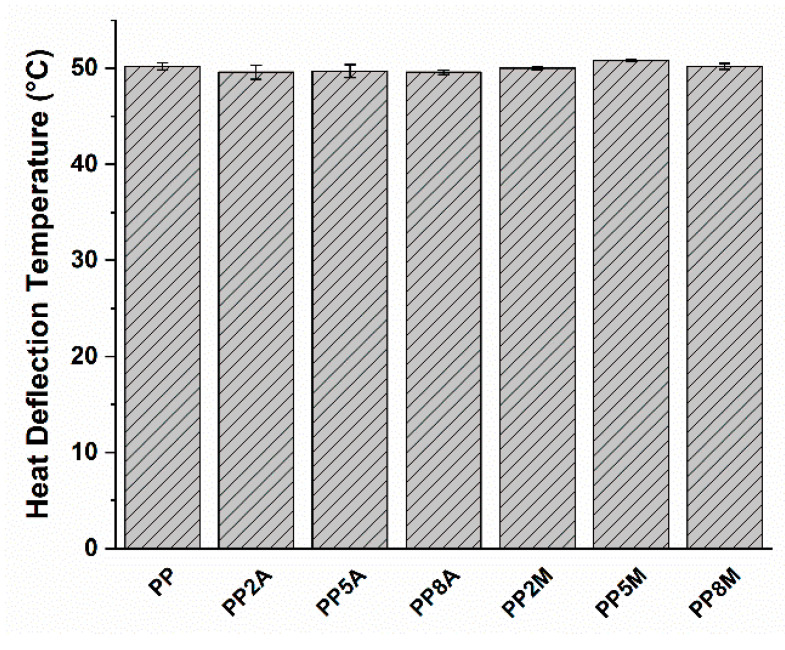
Heat deflection temperature as a function of coupling agent type and amount.

Neat PP exhibited the typical ductile behavior, that was not impaired by the presence of coupling agents. The tensile strength and Young’s modulus of the different matrices were not significantly affected by both coupling agents, as observed in other studies [29], with the exception of a general very limited decrease that can be ascribed to a slight reduction in the total degree of crystallinity, as the coupling agent can alter the regularity of the matrix molecular chains and their packing efficiency [16]. Roux et al. [29] showed that the crystalline structure of the neat PP matrix was modified by the addition of acrylic acid and, to a lesser extent, of maleic anhydride-grafted PP, with the occurrence of smaller crystalline entities but less defined. Watanabe et al. [30] have recently shown that the crystallinity of PP was not significantly influenced by the presence of glass fibers and maleic anhydride-grafted PP. The same conclusions were drawn from the analysis of flexural curves (Figure 3) and related results (Figure 4). It is interesting to note that the contribution of grafted PP to the matrix’s Charpy impact strength (Figure 5) and heat deflection temperature (HDT) (Figure 6) was negligible, thus suggesting that the influence of bulk matrix modification on the mechanical and thermal properties can be ignored.

### 2.2. Effect of Fiber and Coupling Agent Type on PP-based Composites

**Figure 7:**
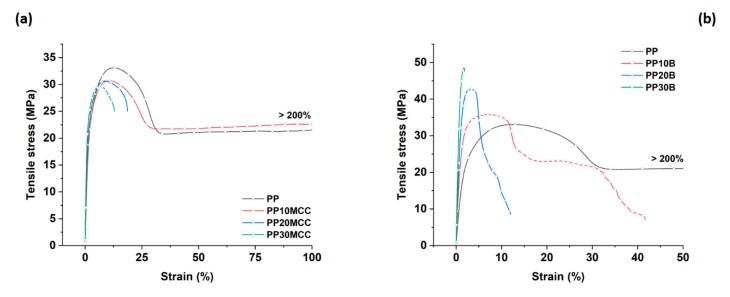
Tensile stress vs. strain curves for PP-based composites reinforced with (**a**) microcrystalline cellulose and (**b**) basalt fibers.

Figure 7 shows the tensile stress vs. strain curves for composites reinforced with microcrystalline cellulose and basalt fibers at different amounts ranging from 10 to 30 wt. %.

**Figure 8:**
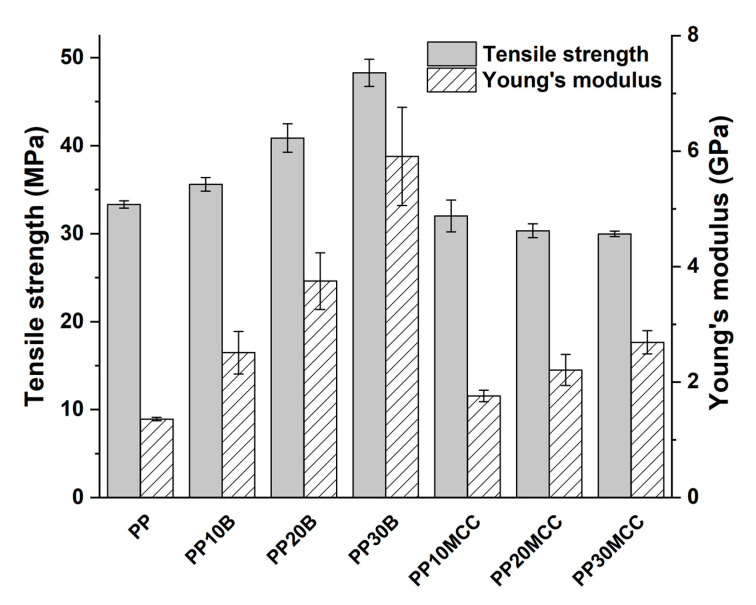
Tensile strength and Young’s modulus as a function of fiber type and amount.

**Figure 9:**
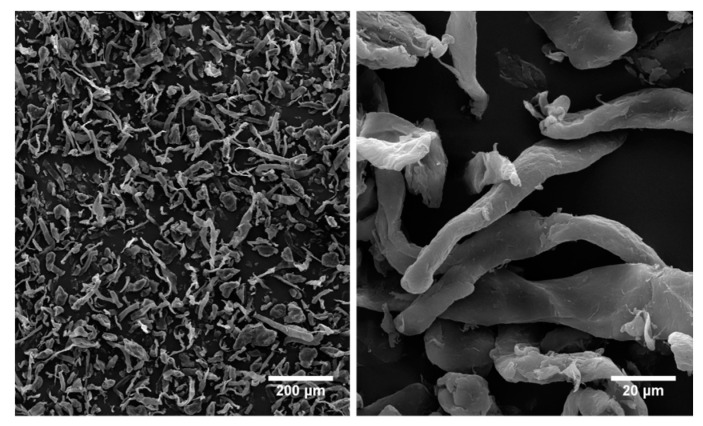
SEM micrographs of as-received microcrystalline cellulose at different magnifications.

The incorporation of microcrystalline cellulose increased the tensile modulus in comparison to the neat PP, but the tensile strength decreased and plateaued with increasing cellulose content, as can be inferred by considering the values summarized in Figure 8. This was quite expected due to the irregular nature and fiber aspect ratio of the microcrystalline cellulose, which featured an intermediate behavior between fiber-like and particle-like structures (Figure 9). In addition, it is worth mentioning that despite the lack of compatibility between hydrophilic cellulose and the hydrophobic PP matrix, the decrease in the tensile strength of composites up to an amount of 30 wt. % is quite limited. On the contrary, the incorporation of basalt fibers increased the tensile modulus and strength of neat PP, due to their higher strength and stiffness compared to microcrystalline cellulose. The tensile strains at failure were remarkably lower than neat PP with increasing fiber content, especially for basalt fiber composites, due to the more brittle nature of basalt fibers compared to cellulose ones [16,31].

**Figure 10:**
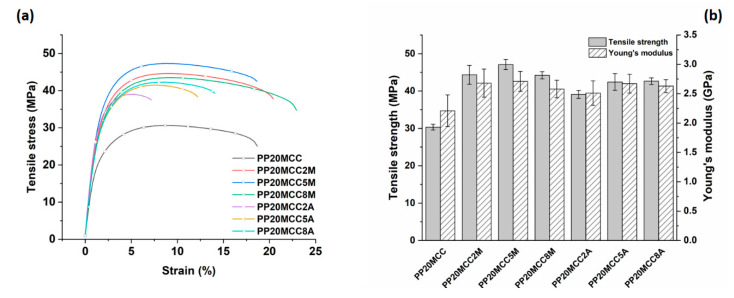
(**a**) Effect of coupling agent on the tensile stress vs. strain curves for PP-based composites reinforced with microcrystalline cellulose, and (**b**) related tensile strength and Young’s modulus as a function of coupling agent amount.

To investigate the effect of the different coupling agents, the weight fraction of basalt and microcrystalline cellulose (MCC) fibers was kept fixed at 20 wt. %, and the amount of coupling agent was varied from 2 to 8 wt. %. Figure 10 shows the effects of coupling agents on the tensile properties of composites reinforced with microcrystalline fibers. The results show the beneficial effects of both coupling agents, with Young’s modulus and tensile strengths that exhibited significant improvements over the non-compatibilized formulation. For both coupling agents, a weight percentage of 5 wt. % provided the highest mechanical properties, though maleic anhydride-grafted polypropylene proved to be the most effective. For composites containing 20 wt. % of MCC and 5 wt. % of MA-g-PP, the tensile strength and Young’s modulus increased by 55% and 22% over the unmodified formulation, respectively, and by 41% and 99% compared to the neat PP. Lesser improvements were achieved with the acrylic acid-grafted polypropylene, whereby the increases in tensile strength and stiffness were 40% and 21% over the unmodified formulation, respectively. The difference in the tensile curves of MA-g-PP and AA-g-PP systems, in particular the higher failure strain of the former, is noteworthy. This behavior can be related to the easier dispersion of the fibers in the extrusion and injection processes, with less fiber agglomeration, which is responsible for stress intensification, causing the early fracture of composites, as well as the suppression of fracture at the fiber/matrix interface [30].

**Figure 11:**
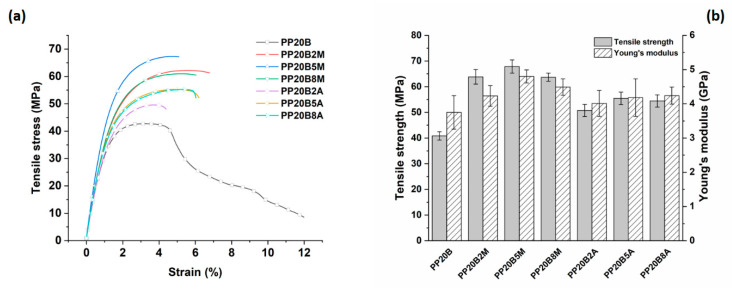
(**a**) Effect of coupling agent on the tensile stress vs. strain curves for PP-based composites reinforced with basalt fibers, and (**b**) related tensile strength and Young’s modulus as a function of coupling agent amount.

Even greater improvements were found with basalt fiber composites, as shown in Figure 11, with a better performance offered by composites modified with MA-g-PP.

In this case, the dependence of tensile properties on the amount of coupling agent highlights the stronger effect of 5 wt. % of MA-g-PP, while the effects of AA-g-PP-modified composites were less evident. Composites containing 20 wt. % basalt fibers and 5 wt. % MA-g-PP exhibited increases in tensile strength and Young’s modulus equal to 66% and 28%, compared to the unmodified formulation, respectively, while the same properties if calculated with relation to neat PP featured remarkable increases of 104% and 253%. Further, for basalt fiber composites, the acrylic acid-modified PP was less effective, with improvements of 36% and 11% for tensile strength and stiffness, respectively.

**Figure 12:**
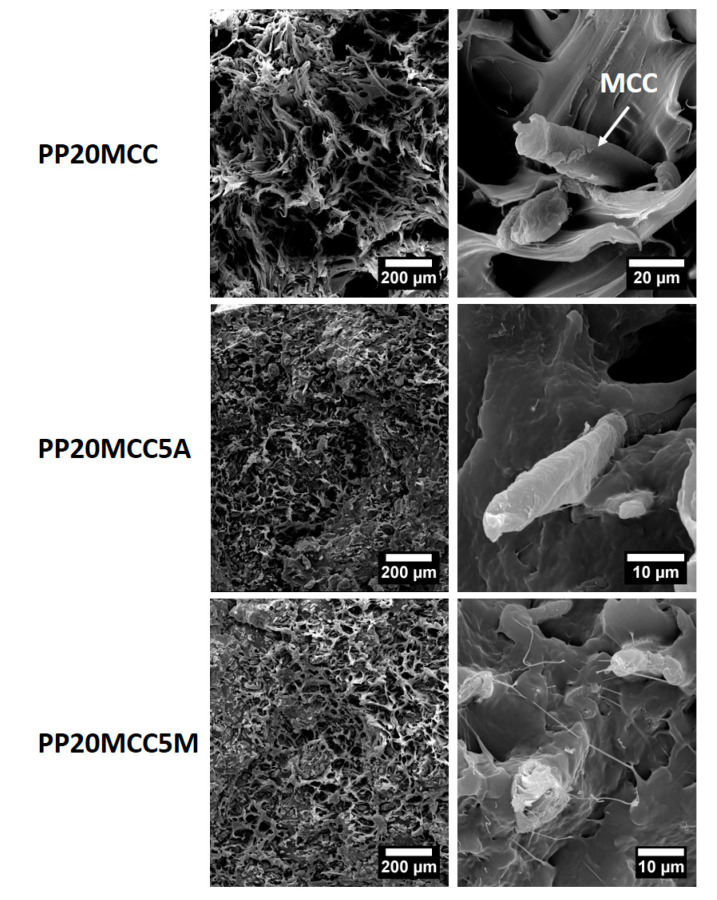
SEM micrographs of microcrystalline cellulose-reinforced PP composites at different magnifications.

**Figure 13:**
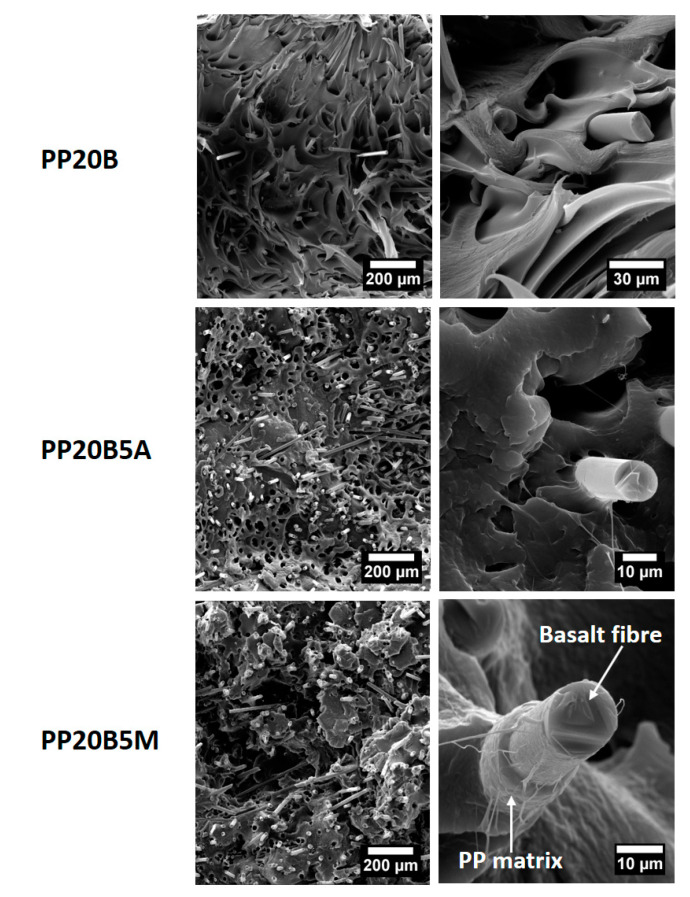
SEM micrographs of basalt fiber-reinforced PP composites at different magnifications.

In general, the stiffness improvements were less significant compared to the strength improvements, which can be explained in terms of the number of coupling sites that lead to a better fiber/matrix adhesion. Tensile strength is related to the post-elastic zone range, where fiber pull-out or fiber failure trigger the final failure irrespective of how stiff the composite is. The bonding between the anhydride group with the hydroxyl groups of cellulose and basalt fibers, accompanied by chain entanglement between MA-g-PP (and AA-g-PP) and PP chains, are the reasons for the generation of a good fiber/matrix interphase and stress transfer [23,32]. Interestingly, the smaller improvements obtained with AA-g-PP for both microcrystalline cellulose and basalt fibers are in line with other studies on recycled newspaper fibers [33] and glass fibers [29]. Despite the higher grafting level compared to MA-g-PP, it is difficult to suppose the occurrence of ester linkages between the acrylic acid and the fiber surface. Both coupling agents in the present study feature low viscosities, therefore differences in their ability to migrate and reach the fiber/matrix interface are unlikely. This is in general an important issue, because it has been shown recently that coupling agents are preferentially distributed around the glass fibers in a glass/PP system [30]. It is suggested that maleic anhydride is in its acid form [30], therefore the fibers have a higher probability of acid–base interactions with the two neighboring carboxylic groups of maleic acid compared, to the single carboxylic group present in the AA-g-PP. This is confirmed by SEM analysis of the fracture surfaces of failed tensile specimens, as reported in Figure 12 and Figure 13 for microcrystalline cellulose- and basalt fiber-reinforced composites, respectively.

For both reinforcements, non-compatibilized formulations displayed a ductile failure at the microscopic scale, while it becomes more brittle after the incorporation of coupling agents, as a result of the lower mobility of the polymer chains. Poor fiber/matrix interfacial adhesion for MCC and basalt fibers can be easily seen, as fiber pull-out and clear gaps between fiber and matrix dominated the fracture surfaces, with fibers scarcely wetted by the matrix. The presence of AA-g-PP increased the interfacial adhesion in both systems, as evidenced by the reduced debonding phenomena at the fiber/matrix interface, though to a lesser extent compared to MA-g-PP. In maleic anhydride compatibilized systems, the fibers are extensively covered by the PP matrix, thus suggesting a remarkably improved interfacial adhesion that is responsible for the better mechanical properties. In basalt fiber-reinforced composites, only the presence of the sizing is insufficient to provide a sound interfacial adhesion, and therefore it is envisaged that the silane component of the commercial sizing reacted with maleic anhydride functional group of the coupling agent, as it is the case for fibers coated with γ-aminopropyl-triethoxysilane, in which the secondary amine group can react with maleic anhydride [29]. In the present case, the actual composition of the commercial sizing is not known, but it is reasonable to suggest the occurrence of a similar mechanism.

**Figure 14:**
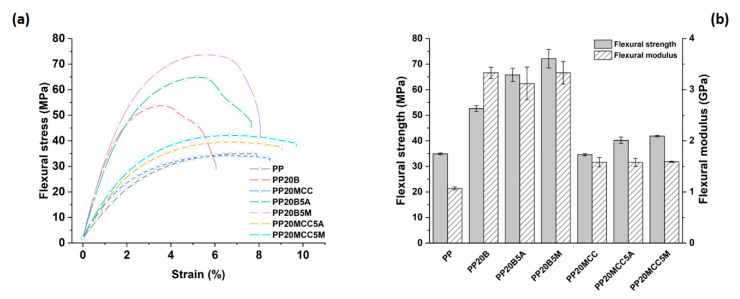
(**a**) Effect of coupling agent on the flexural stress vs. strain curves for PP-based composites reinforced with microcrystalline cellulose and basalt fibers, and (**b**) related flexural strength and modulus as a function of fiber and coupling agent type.

From the tensile results, it appeared that composites loaded with 5 wt. % of coupling agent exhibited the best performance; therefore, these formulations were subjected to further mechanical characterizations based on three-point bending and impact tests. The flexural properties displayed similar trends to those of the tensile properties, as can be inferred from Figure 14, once again confirming the lower reactivity of AA-g-PP compared to MA-g-PP.

**Figure 15:**
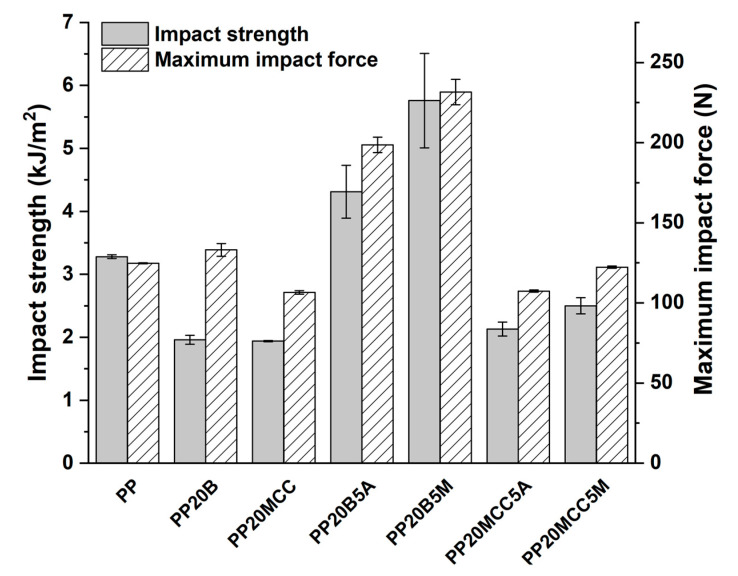
The results of Charpy fracture tests for PP-based composites.

The impact resistance of the samples was also characterized, and is plotted against fiber type and coupling agent in Figure 15. Microcrystalline cellulose acted as a stress concentration, and reduced the impact strength of the composites compared to neat PP, a property only marginally increased with the introduction of coupling agents. The reduced fiber aspect ratio and irregular morphology of MCC resulted in a negligible energy absorption by fiber pull-out mechanism. Similar results were obtained by Batista et al. [31] on cellulose fiber-reinforced PP, and by Jarukumjorn and Suppakarn [34] on sisal/PP composites.

The improved resistance of basalt fibers resulted in higher maximum impact forces, but non-modified systems showed a reduced impact strength compared to neat PP, likely due to the poor interfacial adhesion and low contribution of the fiber pull-out. The incorporation of coupling agents significantly improved the impact strength in the range of 31–76%. The better fiber/matrix adhesion increased the work of fracture of the interphase that resisted crack propagation, thus confirming the higher reactivity of MA-g-PP compared to AA-g-PP. Similar results were found in [21,26].

**Figure 16:**
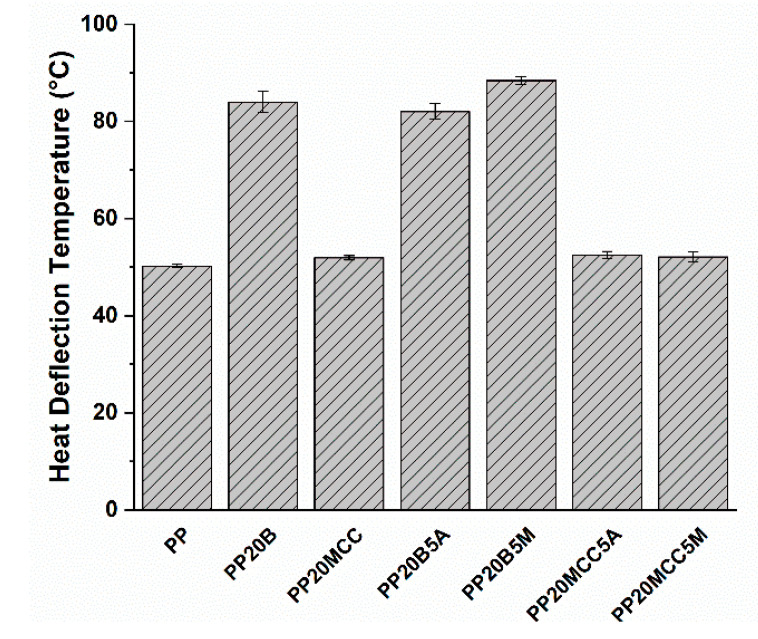
The results of HDT tests for PP-based composites.

The heat deflection temperature of a polymer material is an essential property for the effective design of a product, because it determines the upper stability limit of the material in service without the occurrence of a significant deflection under load and temperature. It is a property that can be largely influenced by the processing conditions of the samples, an issue particularly important when dealing with semi-crystalline polymers [35]. In this case, specimens were manufactured with the same processing parameters, and the results are summarized in Figure 16. HDT can be related to the stiffness of the resulting composites [36], and the incorporation of MCC led to a negligible increase in HDT over the neat PP, as evidenced by the modest increase in stiffness already detected in the three-point bending tests (Figure 14). The introduction of basalt fibers caused, for the compatibilized system, a remarkable increase of 76% over the neat PP [10,12], thus substantially broadening the range of potential applications of these composites compared to neat PP.

### 2.3. Effect of Fiber Hybridization and Coupling Agent on PP-based Composites

**Figure 17:**
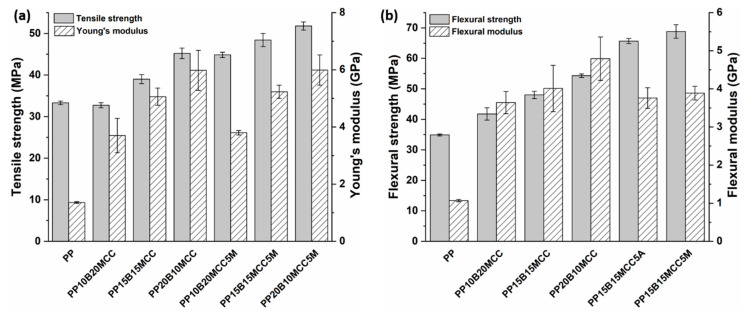
Effect of basalt fiber content on the (**a**) tensile and (**b**) flexural properties of MCC/basalt hybrid PP composites.

The limited mechanical properties, as well as the thermo-mechanical stability of MCC-based composites, led to the development of several formulations whereby MCC and basalt fibers were hybridized in order to obtain a balanced set of properties suitable for automotive applications. The overall fiber weight fraction in hybrid composites was kept constant at 30 wt. %. The variation of tensile and flexural properties with increasing basalt fiber content in MCC/basalt fiber hybrid composites is shown in Figure 17.

Increases in the strength and modulus of MCC-reinforced composites were recorded with the incorporation of basalt fibers due to the better mechanical properties of basalt fibers, as confirmed by other studies based on the hybridization of glass or basalt fibers in natural fiber composites [17,37,38], which had tensile and flexural properties that exhibited a similar trend. MCC/basalt (15/15) hybrid composites with compatibilized matrices showed tensile strength and modulus values that were increased by 45% and 284% in comparison with neat PP, respectively, and by 61% and 94% compared to composites reinforced with 30 wt. % of MCC. As regards the flexural properties, the same hybrid composites featured increased strength and modulus values by 97% and 263% compared to neat PP.

**Figure 18:**
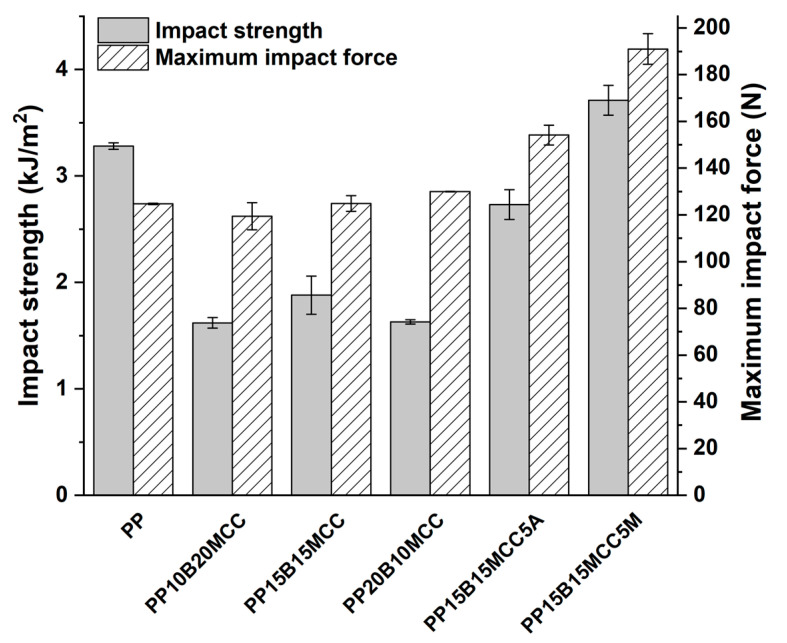
The results of Charpy fracture tests for PP-based hybrid composites.

The results of impact strength are shown in Figure 18.

**Figure 19:**
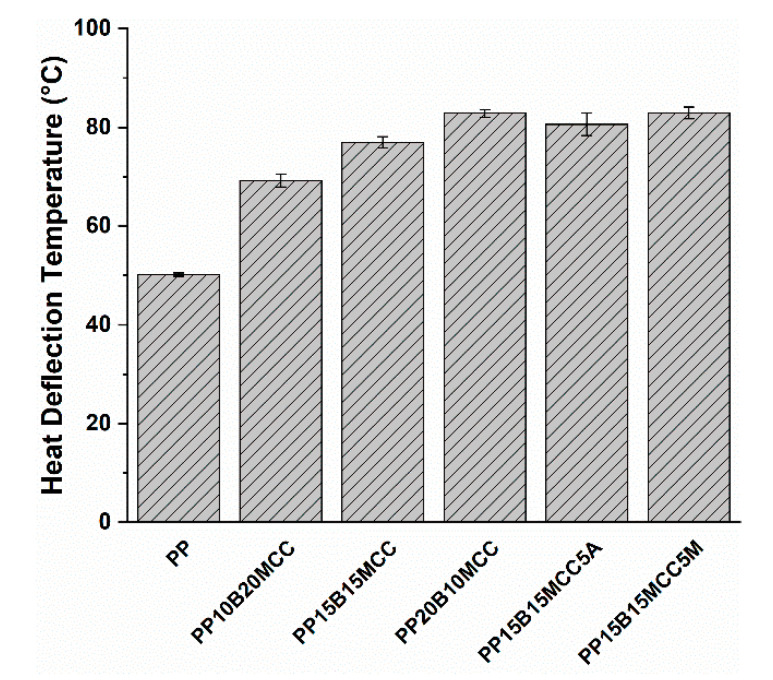
The results of HDT tests for PP-based hybrid composites.

It is interesting to note that hybrid composites did not show any improvement compared to neat PP, once more confirming the important role played by a poor interfacial adhesion and the limited effectiveness of cellulose in improving the impact strength [1]. Only when the MA-g-PP was added did the increased interfacial adhesion allow the MCC/basalt (15/15) hybrid composites to enhance the impact strength by 13% in comparison with neat PP. The limited thermomechanical stability of MCC-based composites was improved by the incorporation of basalt fibers, as can be seen in Figure 19. The optimal formulation containing a basalt fiber to MCC ratio of 15/15 and 5 wt. % of MA-g-PP increased the HDT by 65% compared to neat PP.

The results of the present study on hybrid composites compare quite favorably with those reported in reference [1], where the authors investigated the combination of glass and cellulose fibers in polyamide 6/polypropylene blends specifically designed for automotive applications. In reference [31], the authors addressed the mechanical properties of different hybrid formulations based on inorganic reinforcements (long and short glass fibers, mica), cellulose and PP matrices for automotive applications. Raw materials were supplied as master-batch pellets from different commercial manufacturers. As a matter of comparison, mechanical results are summarized in Table 1 along with one formulation investigated in the present work. It is evident that basalt fibers in combination with cellulose fibers in a PP matrix are competitive with the systems from reference [31], which were specifically suggested for body interior (e.g., console substrate, wiring harness) and under-the-hood (e.g., battery and power distribution box covers) applications.

## 3. Materials and Methods

### 3.1. Raw Materials

A polypropylene ISPLEN PP094 supplied by Repsol (Milan, Italy) was used as matrix, while chopped basal fibers (B), provided by Incotelogy GmbH (Pulheim, Germany), exhibited an average diameter of 13 μm with a nominal length of 3.2 mm. These fibers were supplied with a silane coating suitable for PP. Microcrystalline cellulose (MCC) (average particle size 51 μm) was supplied by Sigma Aldrich. Two different coupling agents were used: (i) a maleic anhydride-modified PP, Polybond^®^ 3200 by Addivant Corporation (Danbury, CT, USA), characterized by a maleic anhydride content in the range 0.8–1.2% and a melt flow rate @190 °C, 2.16 kg equal to 115 g/10 min; (ii) an acrylic acid-modified PP, Polybond^®^ 1103 by Addivant Corporation (Danbury, CT, USA), characterized by an acrylic acid content in the range 5.5–6.5% and a melt flow rate @230 °C, 2.16 kg equal to 380 g/10 min.

### 3.2. Processing

Specimens for the mechanical and thermal characterizations were manufactured by extrusion and injection molding. Prior to extrusion, materials were dried overnight at 80 °C. Extrusion was carried out in a co-rotating twin-screw extruder (Thermo Scientific Process 11, Thermo Fisher Scientific, Waltham, MA, USA) and the tensile, flexural and impact specimens were obtained by injection molding (Haake MiniJet II Pro, Thermo Fisher Scientific). The temperature profile (8 zones) inside the extruder was as follows (from feed to die): 170-180-180-190-200-200-290-190 °C. The screw speed was set at 200 rpm. The mold during injection molding was maintained at 35 °C, while the loading cylinder was heated at 190 °C. The injection procedure consisted of two steps: a first injection step at a pressure of 450 bar for 10 s, and a post injection step at 60 bar for an additional 10 s. All the formulations along with the characterization techniques used for each formulation are listed in Table 2.

### 3.3. Mechanical Characterization of Composites

Tensile tests were performed on a Zwick/Roell Z010 (Ulm, Germany) in displacement control with a cross-head speed of 10 mm/min with type 1BA samples (l_0_ = 30 mm) in accordance with UNI EN ISO 527-2. The strain was measured with a contacting extensometer, and at least five tests for each material formulation were carried out.

Three-point bending tests were performed in accordance with ISO 178 on a Zwick/Roell Z010 with a cross-head speed of 5 mm/min and a support span length of 64 mm. Specimens had dimensions of 80 × 10 × 4 mm. Strain was measured with a displacement transducer in contact with the samples, and at least five tests for each material formulation were carried out.

Charpy impact strength was determined in accordance with ISO 179-2 in an edgewise condition. The notched specimens of 80 × 10 × 4 mm size had a notch type A and a span of 62 mm was used. The tests were performed with a motorized pendulum CEAST 9050 (Pianezza, Italy) by using an impact energy of 0.540 J and an impact velocity of 1.08 m/s. At least five tests for each material formulation were carried out.

### 3.4. Thermal Characterization

The temperature of deflection under load (HDT) was performed in an oil bath by means of an Instron HV6X HDT tester (Pianezza, Italy) in accordance with ISO 75-2. Specimens of dimensions 80 × 10 × 4 mm were subjected to Method A (1.8 MPa–120 °C/h) with a span of 64 mm. At least three tests for each material formulation were carried out.

### 3.5. Morphological Characterization

Composite fracture surfaces were investigated by scanning electron microscopy (SEM), using a FE-SEM Mira3 by Tescan (Brno, Czech Republic). To prevent charging, the specimens were sputter coated with gold prior to analysis.

## 4. Conclusions

In this study, the hybridization of microcrystalline cellulose with basalt fibers in a PP matrix was exploited for automotive applications. Composites were processed by extrusion and injection molding with an overall reinforcement amount of 30 wt. %. In an attempt to increase the poor interfacial adhesion with a hydrophobic matrix, the effects of two coupling agents, with maleic anhydride and acrylic acid functionalities, at different quantities on the mechanical and thermal properties of the composites were investigated. Acrylic acid-grafted PP proved to be less effective than maleic anhydride-grafted PP, irrespective of fiber type. A content of 5 wt. % was able to promote remarkable improvements in tensile, flexural, impact and HDT properties. Results from the tensile, flexural and notched Charpy impact tests showed a decrease in mechanical properties with increasing cellulose content, but composites with an optimum amount of microcrystalline cellulose exhibited adequate properties, suitable to limiting the presence of inorganic reinforcements in many automotive applications. In particular, the compatibilized MCC/basalt (15/15) hybrid composites enhanced tensile strength and modulus, flexural strength and modulus, and impact strength and HDT by 45% and 284%, 97% and 263%, and 13% and 65% in comparison with neat PP, respectively. This work provides guidelines for designing hybrid formulations based on natural fibers of mineral and plant origin meant for automotive applications, leading to weight and cost savings with enhanced sustainability.

## Figures and Tables

**Table 1:** Summary of mechanical properties of hybrid composites in comparison with literature results (LGF = long glass fibers; SGF = short glass fibers. Relative fiber proportions are expressed in wt. %).

Formulation	Tensile Strength (MPa)	Tensile Modulus (GPa)	Flexural Strength (MPa)	Flexural Modulus (GPa)	Reference
PP15B15MCC5M	48.4 ± 1.6	5.2 ± 0.2	68.8 ± 2.2	3.9 ± 0.2	This study
LGF/Cellulose (20/10)	59.7	5.2	−	4.3	[31]
LGF/Cellulose (15/15)	51.6	4.6	−	4.0	[31]
LGF/Cellulose (10/20)	45.7	3.5	−	3.6	[31]
SGF/Cellulose (20/10)	53.4	5.5	−	4.1	[31]
SGF/Cellulose (15/15)	48.1	4.9	−	3.8	[31]
SGF/Cellulose (10/20)	43.2	4.5	−	3.4	[31]
(SGF/Mica)/Cellulose (20/10)	48.0	4.9	−	3.7	[31]
(SGF/Mica)/Cellulose (15/15)	44.8	4.6	−	3.5	[31]
(SGF/Mica)/Cellulose (15/15)	42.5	4.4	−	3.3	[31]

“−” stands for property not measured.

**Table 2:** Summary of PP-based composites and related characterization techniques.

Formulation	PP (wt. %)	MA-g-PP (wt. %)	AA-g-PP (wt. %)	Basalt Fibers (wt. %)	MCC (wt. %)	Tensile Test	Flexural Test	Impact Test	HDT
PP	100	−	−	−	−	x	x	x	x
PP2A	98	−	2	−	−	x	x	x	x
PP5A	95	−	5	−	−	x	x	x	x
PP8A	92	−	8	−	−	x	x	x	x
PP2M	98	2	−	−	−	x	x	x	x
PP5M	95	5	−	−	−	x	x	x	x
PP8M	92	8	−	−	−	x	x	x	x
PP10B	90		−	10	−	x	−	−	−
PP20B	80	−	−	20	−	x	x	x	x
PP30B	70	−	−	30	−	x	−	−	−
PP20B2M	78	2	−	20	−	x	−	−	−
PP20B5M	75	5	−	20	−	x	x	x	x
PP20B8M	72	8	−	20	−	x	−	−	−
PP20B2A	78	−	2	20	−	x	−	−	−
PP20B5A	75	−	5	20	−	x	x	x	x
PP20B8A	72	−	8	20	−	x	−	−	−
PP10MCC	90	−	−	−	10	x	−	−	−
PP20MCC	80	−	−	−	20	x	x	x	x
PP30MCC	70	−	−	−	30	x	−	−	−
PP20MCC2M	78	2	−	−	20	x	−	−	−
PP20MCC5M	75	5	−	−	20	x	x	x	x
PP20MCC8M	72	8	−	−	20	x	−	−	−
PP20MCC2A	78	−	2	−	20	x	−	−	−
PP20MCC5A	75	−	5	−	20	x	x	x	x
PP20MCC8A	72	−	8	−	20	x	−	−	−
PP10B20MCC	70	−	−	10	20	x	x	x	x
PP15B15MCC	70	−	−	15	15	x	x	x	x
PP20B10MCC	70	−	−	20	10	x	x	x	x
PP10B20MCC5M	65	5	−	10	20	x	−	−	−
PP15B15MCC5M	65	5	−	15	25	x	x	x	x
PP20B10MCC5M	65	5	−	20	10	x	−	−	−
PP15B15MCC5A	65	−	5	15	15	−	x	x	x

“−” stands for “not available”, while “x” stands for “available”.
